# Data‐Driven Machine Learning–Based Forecasting of Dengue in Bangladesh: Supporting Digital Health Approaches for Early Warning

**DOI:** 10.1002/hsr2.72147

**Published:** 2026-03-19

**Authors:** Arman Hossain Chowdhury

**Affiliations:** ^1^ Department of Statistics Begum Rokeya University Rangpur Bangladesh

**Keywords:** disease trend, epidemiology, health policy, infectious disease, public health

## Abstract

**Background & Aims:**

Dengue is a significant vector‐borne disease that has severely impacted public health in Bangladesh, underscoring the growing importance of digital health in enhancing surveillance and prevention. Understanding its trends and future estimates is crucial for improving early prevention strategies. This study aimed to model trends and select the best model to forecast dengue cases in Bangladesh for the next 5 years to aid digital health early warnings.

**Methods:**

The monthly dengue case data (January 2000 to December 2023) were obtained from the Directorate General of Health Services (DGHS). An autoregressive integrated moving average (ARIMA) and eXtreme gradient boosting (XGBoost) model were employed to analyze the data. The root mean square error (RMSE), mean absolute error (MAE), and mean absolute scaled error (MASE) were used to evaluate the model's performance.

**Results:**

From 2000 to 2023, Bangladesh reported 565,890 confirmed dengue cases, marking a sharp peak in 2023 with 321,179 cases, alongside high incidence rates of 194.47, and a lowest count in 2014 with only 375 cases. A distinct seasonal trend was observed, with cases rising in June, peaking in August, and declining by October. To identify the most suitable model, both ARIMA and XGBoost were evaluated. Performance metrics indicated that XGBoost outperformed ARIMA (RMSE = 0.63, MAE = 0.54, MASE = 0.39) in predicting dengue cases. Feature importance analysis showed that recent dengue incidence, especially at lag 1, was the most prominent predictor, with further impact from longer‐term and seasonal recurrence patterns. Consequently, XGBoost was employed to forecast future incidences, projecting that dengue cases may range from 35,297 to 330,242 between 2024 and 2028, suggesting a potential rise in future outbreaks.

**Conclusion:**

The findings underscore the urgent need to strengthen early warning systems and leverage digital health tools to manage the escalating dengue threat. Integrating machine learning models into public health strategies can enhance predictive accuracy and inform targeted interventions. Future research should consider additional factors, such as climate and urbanization, to refine projections and support effective disease management.

## 1 Introduction

Dengue, a Spanish word that means fastidiousness [1], is the fastest‐spreading mosquito‐borne infectious disease and a major public health concern, particularly in tropical and subtropical countries like Bangladesh [2]. It is caused by four serotypes of the dengue virus (DENV 1‐4) [3] and transmitted by Aedes mosquitoes. Recent research estimates that 3.9 billion people worldwide are at risk of contracting dengue fever, with the majority of cases concentrated in South and Southeast Asia, accounting for 70% of the global burden [2]. Each year, the World Health Organization (WHO) reports between 50 and 100 million cases of dengue fever worldwide, resulting in approximately 20,000 recorded deaths [4, 5].

Bangladesh recorded its inaugural dengue epidemic in 1964 [6], with subsequent outbreaks occurring periodically, including a significant epidemic in 2000 that resulted in 5551 reported cases and 93 fatalities [7]. This particular epidemic in 2000 was likely instigated by the introduction of a DENV strain, potentially originating from Thailand [8]. The 2019 outbreak was the most severe to date, with over 112,000 reported cases and 129 deaths, mostly concentrated in Dhaka [6]. Between January 1 and August 7, 2023, the Ministry of Health and Family Welfare of Bangladesh reported 69,483 laboratory‐confirmed dengue cases and 327 related fatalities, resulting in a case fatality rate (CFR) of 0.47% [9]. These repeated epidemics highlight the pressing need for timely surveillance, effective public health responses.

Climatic conditions, such as rainfall, humidity, and temperature variations, play a significant role in dengue transmission [10]. However, the complexity of these environmental interactions and the nonlinear nature of outbreak patterns make accurate prediction challenging. This underscores the need to leverage digital health tools, such as real‐time disease surveillance systems and data‐driven forecasting models, to better predict outbreaks and guide interventions. Such tools are increasingly being used globally to monitor and manage infectious diseases.

To better manage and predict future outbreaks, accurate forecasting of dengue cases is essential. Time series models, such as the autoregressive integrated moving average (ARIMA) model, have been widely applied to infectious diseases, including COVID‐19 [11, 12], hemorrhagic fever [13], influenza [14], malaria [15], and tuberculosis [16] due to their ability to capture trends and temporal dependencies [11, 12]. Yet, ARIMA models often struggle with nonlinear patterns, limiting their predictive accuracy for complex outbreaks. Machine learning models, such as the eXtreme gradient boosting (XGBoost) model, handle these types of problems better through methods like feature transformation and ensemble approaches [17]. The XGBoost model has outperformed others in many previous studies and achieved great accuracy [12, 18, 19]. Despite its proven capabilities, XGBoost has not yet been applied to long‐term univariate dengue prediction in Bangladesh.

Given the critical role of time series forecasting in understanding epidemic spread, the objective of this study is to model the overall trend and identify the best model to project dengue cases in Bangladesh for the next 5 years. By providing actionable forecasts, the findings will support policymakers in strengthening prevention measures, optimizing resource allocation, and integrating digital health solutions to improve early warning systems and reduce the burden of dengue.

## 2 Methods

### 2.1 Data Collection

Monthly confirmed dengue case data were collected from the Directorate General of Health Services (DGHS), spanning from January 2000 to December 2023 [20]. The yearly population data used to compute incidence rates were obtained from the World Bank Group website [21]. To compute the incidence rates by district in 2023, the population data for each district were collected from the population and housing census (PHC‐2022) [22]. Before modeling, the entire dataset was split into a training set and a testing set. The training set includes 90% of the data (from January 2000 to July 2021), while the testing set includes the remaining 10% (from August 2021 to December 2023).

### 2.2 Statistical Analyses

**Figure 1:**
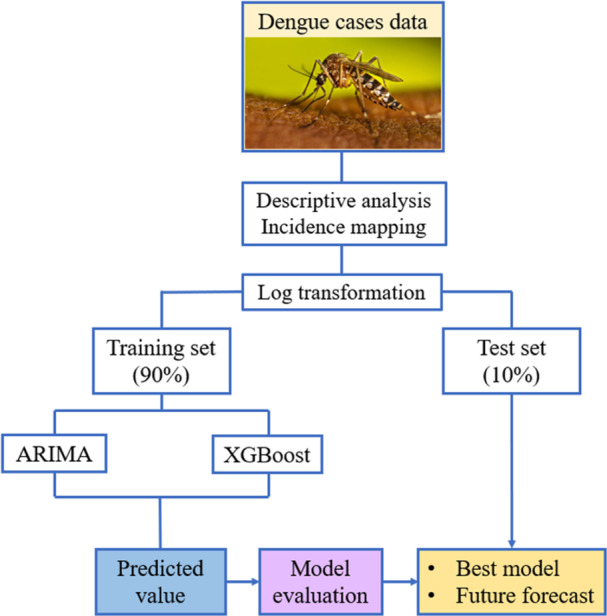
Diagram of the proposed methodology.

The analysis began with aggregating monthly dengue cases into yearly counts, followed by a descriptive analysis to determine the minimum, maximum, mean, and standard deviation of cases. Incidence rates per 100,000 population [23, 24] were then calculated for each year and each district of Bangladesh for 2023. Subsequently, the ARIMA model was developed using the monthly dengue dataset, encompassing various stages, including log transformation, the Augmented Dickey‐Fuller test, seasonal decomposition, and so on. The XGBoost model was then fitted by tuning its several parameters, and the model was selected at its best tune. Future projections for the next 5 years were produced using the top‐performing model (Figure 1). Since the “forecastxgb” framework does not inherently offer prediction intervals, 95% intervals were created afterward by assuming the forecast errors follow a normal distribution. Specifically, the root mean squared error (RMSE) from the test set served as an estimate of forecast uncertainty, and the intervals were computed as the point forecast ±1.96 times the RMSE on the log scale, then transformed back to the original scale. All analyses were performed using RStudio (Version 4.4.0) [25] with a range of R packages, including ‘readxl', “tidyverse,” “tseries,” “forecast,” “forecastxgb,” and “ggplot2.” To create the geospatial map, this study employed the “ggplot2,” “maps,” and “sf” packages, utilizing publicly available shapefile data from the Global Administrative Areas Database (GADM) [26].

### 2.3 ARIMA Model

The ARIMA model, developed by Box and Jenkins in 1976, is a technique designed for the analysis and prediction of time series data [18]. ARIMA components are categorized into three parts: AR (autoregressive), I (integrated), and MA (moving average) [27]. The parameters of this model are as follows: ARIMA (*p*, *d*, *q*). In the ARIMA model, the letter *p* represents the order of the autoregressive (AR) component, *d* indicates the differencing order, and *q* signifies the order of the moving average (MA) component [11]. The AR component simulates the relationship between an observation and its prior values [28]. The MA component simulates how present values are impacted by random variations in the past. Differencing (I) eliminates non‐stationarity or trends from the data. By taking into consideration recurring seasonal patterns, seasonal ARIMA goes beyond this, which is especially helpful for illnesses that exhibit seasonal fluctuation, such as dengue. For more information on ARIMA and SARIMA models, refer to the supplement.

### 2.4 XGBoost Model

The robust machine learning technique XGBoost (eXtreme Gradient Boosting) is usually utilized for problems involving regression or classification and structured data [12, 29]. It is a decision tree‐based ensemble machine learning approach where each tree is trained to correct the errors of the previous trees to improve accuracy. By combining the results from multiple individual trees through an internal aggregation method, it can produce accurate predictions [18]. XGBoost was first introduced by Tianqi Chen and Carlos Guestrin in 2011, and it has since been refined and enhanced by several researchers for further studies [11, 18]. Boosting is a technique that enhances machine learning models by combining multiple weak prediction models into a single strong model with improved accuracy. Achieving reliable prediction often requires integrating several models while maintaining reasonable parameter settings. When dealing with large or complex datasets, multiple iterations may be needed to attain the desired accuracy. XGBoost is particularly effective in handling such scenarios [30]. In this study, the model parameters were adjusted to reduce prediction error while avoiding overfitting, using lagged dengue case counts as input characteristics. Future dengue cases were then predicted using the generated model. More information regarding xgboost can be found in the supplement.

### 2.5 Model Assessment

The primary metric for evaluating the model is its accuracy, which measures how closely the predicted values match the actual values [12]. There are various methods to assess model accuracy. In this study, we used three different metrics: Mean Absolute Error (MAE), Root Mean Square Error (RMSE), and Mean Absolute Scaled Error (MASE). MAE calculates the average absolute prediction error, serving as the arithmetic mean of the discrepancies between predicted and actual values. Meanwhile, RMSE denotes the square root of the average squared differences between the predictions and the real values [19]. Conversely, MASE serves to assess prediction models, especially in time series analysis. It remains stable and reliable, even when actual values are zero or close to zero. These metrics can be mathematically defined as follows:

(1) $$\mathrm{MAE}=\frac{1}{n}\sum_{i=1}^{n}|\hat{y_{i}}-y_{i}|$$

(2) $$\mathrm{RMSE}=\sqrt{\frac{1}{n}\sum_{i=1}^{n}{\left(\hat{y_{i}}-y_{i}\right)}^{2}}$$

(3) $$\mathrm{MASE}=\frac{\mathrm{MAE}}{\mathrm{MA}E_{\mathrm{naive}}}$$

Where *n* is the number of observations, ${\hat{y}}_{i}$ is the estimated number of dengue cases and $y_{i}$ is the true number of dengue cases, and ${\hat{y}}_{i}-y_{i}$ represents the residual number [11]. Here, the MAE_naive_ is the benchmark for the MASE model.

## 3 Results

From 2000 to 2023, 565,890 confirmed dengue cases were reported in Bangladesh. The highest number of cases, 321,179, occurred in the year 2023, while the lowest, 375, was recorded in the year 2014. The mean number of cases was 26,764.92 (SD = 31,932.96) in 2023. The highest incidence rate, 194.47 per 100,000 population, was observed in 2023, compared to the lowest rate of 0.28 in 2010 (Table 1).

**Table 1:** Summary statistics of monthly dengue cases and incidence rates in Bangladesh from 2000 to 2023.

Year	Min	Max	Mean ± SD	Total	IR
2000	0	2290	462.58 ± 684.63	5551	4.30
2001	0	655	202.50 ± 255.76	2430	1.85
2002	0	3281	519.33 ± 1004.11	6232	4.65
2003	0	372	40.50 ± 108.19	486	0.36
2004	0	1261	327.83 ± 460.57	3934	2.83
2005	0	337	87.33 ± 132.56	1048	0.74
2006	0	972	183.33 ± 314.48	2200	1.54
2007	0	179	38.83 ± 66.92	466	0.32
2008	0	475	96.08 ± 161.20	1153	0.79
2009	0	188	39.50 ± 71.66	474	0.32
2010	0	183	34.08 ± 66.38	409	0.28
2011	0	800	113.25 ± 233.49	1359	0.90
2012	0	262	55.92 ± 81.71	671	0.44
2013	0	495	145.75 ± 177.14	1749	1.14
2014	0	82	31.25 ± 33.27	375	0.24
2015	0	965	263.50 ± 375.09	3162	2.00
2016	3	1544	505.00 ± 588.63	6060	3.79
2017	36	512	230.75 ± 165.27	2769	1.71
2018	7	3087	845.67 ± 1064.10	10,148	6.20
2019	17	52,636	8443.67 ± 15,226.60	101,324	61.22
2020	10	546	99.42 ± 148.17	1193	0.71
2021	3	7841	2369.08 ± 3054.99	28,429	16.79
2022	20	21,932	5257.42 ± 8157.92	63,089	36.85
2023	111	79,598	26,764.92 ± 31932.96	321,179	194.47

Abbreviations: IR, incidence rates per 100,000 population; Max, maximum; Min, minimum; SD, standard deviation.

**Figure 2:**
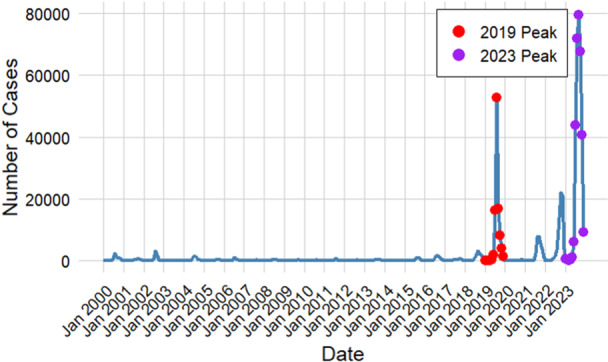
Time series plot of monthly dengue cases in Bangladesh from 2000 to 2023, emphasizing the epidemic years 2019 and 2023.

Dengue became an epidemic in Bangladesh in 2019 and reached its peak in 2023, with 101,324 and 321,179 cases reported, respectively. These 2 years represent the highest levels of dengue incidence in the country (Figure 2). Apart from these peaks, the incidence of dengue cases in other years appears relatively stable, with the case numbers showing a minimal and consistent pattern, almost resembling a straight line, indicating periods of lower incidence.

**Figure 3:**
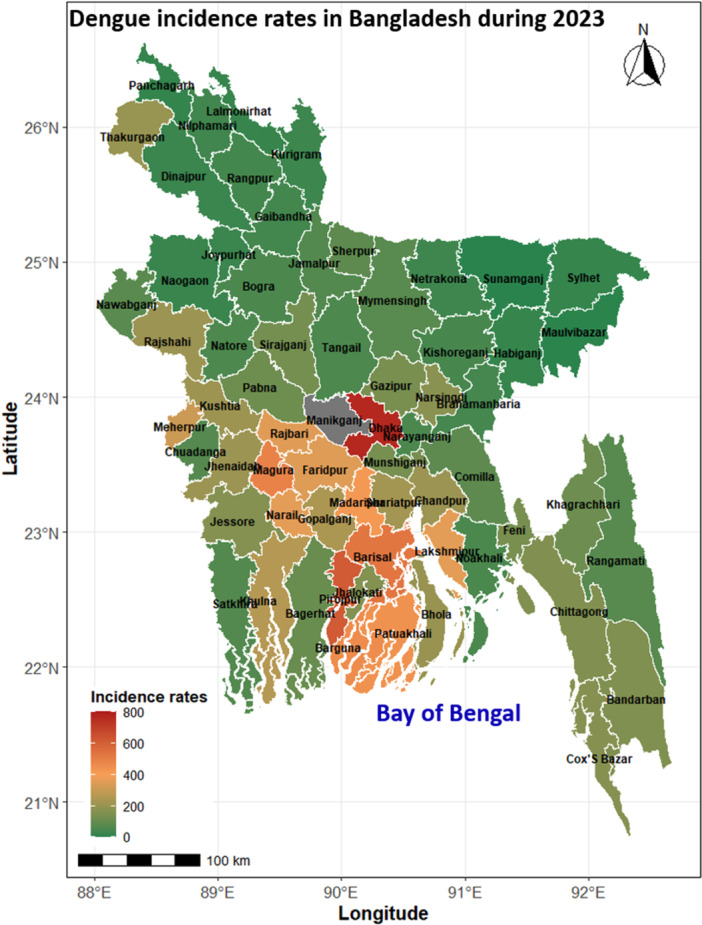
Dengue incidence per 100,000 population across Bangladesh in 2023.

The year 2023 has witnessed an unprecedented surge in dengue outbreaks throughout Bangladesh, representing one of the highest recorded incidence rates in recent years. Several districts have experienced alarming increases in dengue cases, with Manikganj, Dhaka, Pirojpur, Barisal, and Magura reporting notably elevated incidence rates (Table S1). This significant rise indicates an intensification of dengue transmission dynamics (Figure 3).

**Figure 4:**
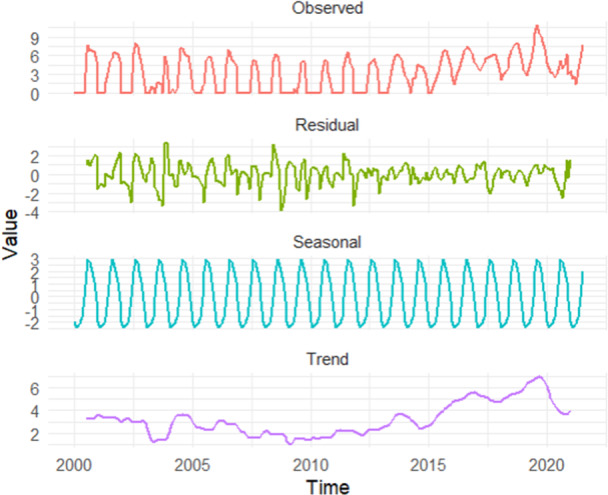
Seasonal decomposition of the monthly dengue data.

The presence of two notable peaks in 2019 and 2023 suggests that applying a log transformation could help stabilize the data and address outliers. Analyzing the decomposed transformed data reveals a clear seasonal pattern, even with the prominent peaks in 2019 and 2023 (Figure 4).

**Figure 5:**
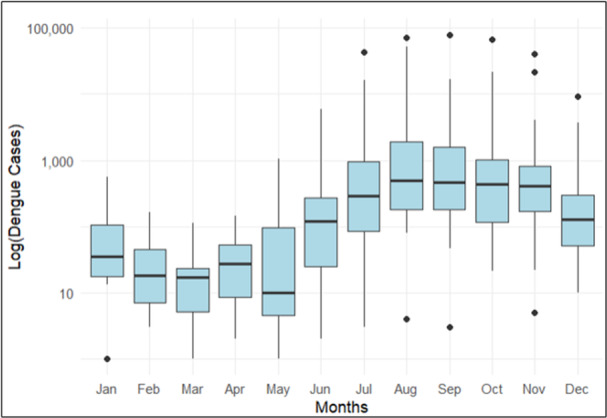
Box plot of monthly cases of dengue in Bangladesh from 2000 to 2023.

In terms of seasonality, the results highlight a clear pattern in the occurrence of dengue cases, with numbers rising significantly in the middle of the year. Specifically, dengue cases begin to increase in June, reach their highest point in August, and then gradually decline starting in October, indicating that August is particularly prone to outbreaks (Figure 5).

Due to the peaks and variations in cases, this study applied a log transformation to stabilize the data. This transformation rendered the data stationary without requiring differencing (Figure S1), as confirmed by the ADF test (*p* < 0.01), a prerequisite for fitting an ARIMA model. However, because the data exhibited a seasonal effect, seasonal differencing was applied, and the data became stationary after the first seasonal differencing, as confirmed by the ADF test (*p* < 0.01). Thus, the parameters *d* and *D* are 0 and 1, respectively. To determine the remaining parameters of the ARIMA model, ACF and PACF plots were constructed. The ACF plot showed a significant peak at lag 0, suggesting that the non‐seasonal MA component may be 0, and a significant peak at lag 24, indicating that the seasonal MA component may be 2. In the PACF plot (Figure S2), significant spikes at lag 1 suggested that the non‐seasonal AR component may be 1, and a significant peak at lag 0 indicated that the seasonal AR component may be 0. Therefore, the maximum values for *p*, *q*, *P*, and *Q* are 1, 0, 0, and 2, respectively.

**Figure 6:**
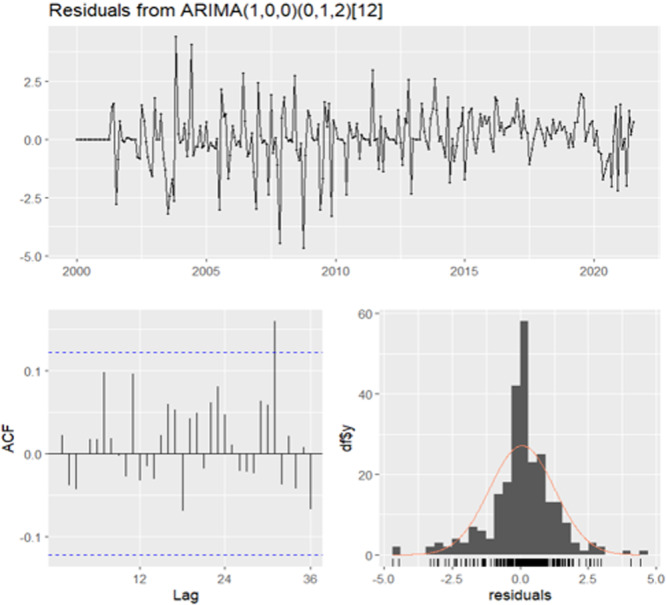
A time series plot of the residuals, along with the associated autocorrelation function (ACF) diagram and a histogram, for the ARIMA (1,0,0) × (0,1,2)_12_ model.

Based on the above information, the ARIMA model was fitted using the “auto.arima” function, which evaluated all possible models. The model ARIMA(1,0,0) × (0,1,2)_12_ was selected based on the lowest Corrected Akaike's Information Criterion (AICc) value (Table S2). Residual diagnostics confirmed the adequacy of the fitted model. The Ljung–Box test (Q* = 1.52, *df* = 22, *p* = 1.00) showed no significant autocorrelation, indicating that the residuals were independent and the model captured the temporal pattern effectively. Subsequently, the residuals were analyzed using residual plots, the ACF of the residuals, and a residual histogram, which indicated a normal distribution (Figure 6). Therefore, the ARIMA(1,0,0) × (0,1,2)_12_ model was deemed significant. The parameters of the model are presented in Table 2.

**Table 2:** Estimated parameters of the ARIMA (1,0,0) × (0,1,2)_12_ model for dengue prediction.

Parameters	Coefficients	SE
ar1	0.70	0.05
sma1	−0.79	0.06
sma2	0.17	0.07
AICc	816.08	

Abbreviations: AICc, Corrected Akaike's Information Criteria; ar, autoregressive; SE, standard error; sma, Seasonal Moving average.

The XGBoost model was fitted using the “forecastxgb” package, which preserves the temporal order of observations to prevent information leakage. A 10‐fold blocked time‐series cross‐validation (nfold = 10) was applied within the “xgbar()” function, ensuring that training and validation sets respected the chronological sequence. The model incorporated 24 lagged observations (lag1–lag24) as predictors to capture temporal dependence, with trend adjustment through differencing and seasonal adjustment using monthly dummy variables (seas_method = “dummies”). A grid of boosting rounds (nrounds = 10–50) was tested, and the optimal number (nrounds = 23) was selected based on the lowest cross‐validation error. All remaining XGBoost hyperparameters, such as tree depth, learning rate, and subsampling rate, were kept at their default package settings to maintain model stability and prevent overfitting. The final tuned model was then fitted to generate the forecasts (Table S3). The resulting curves, showing the actual, fitted, and forecasted dengue cases in Bangladesh, based on both the ARIMA(1,0,0) × (0,1,2)_12_ and XGBoost models, are visually represented in Figure S3 in the supplement.

While both models demonstrate low errors based on the evaluation metrics RMSE, MAE, and MASE, the XGBoost model outperforms the ARIMA model on the test set (Table S4), showcasing its superior ability to predict monthly dengue cases in Bangladesh (Table 3).

**Table 3:** Assessment of ARIMA and XGBoost model parameters for dengue cases in Bangladesh.

Models	Dataset	RMSE	MAE	MASE
ARIMA	*Training*	1.19	0.79	0.57
	*Testing*	2.52	2.12	1.53
XGBoost	*Training*	1.53	1.16	0.84
	*Testing*	0.63	0.54	0.39

Abbreviations: MAE, mean absolute error; MASE, mean absolute scaled error; RMSE, root mean square error.

**Figure 7:**
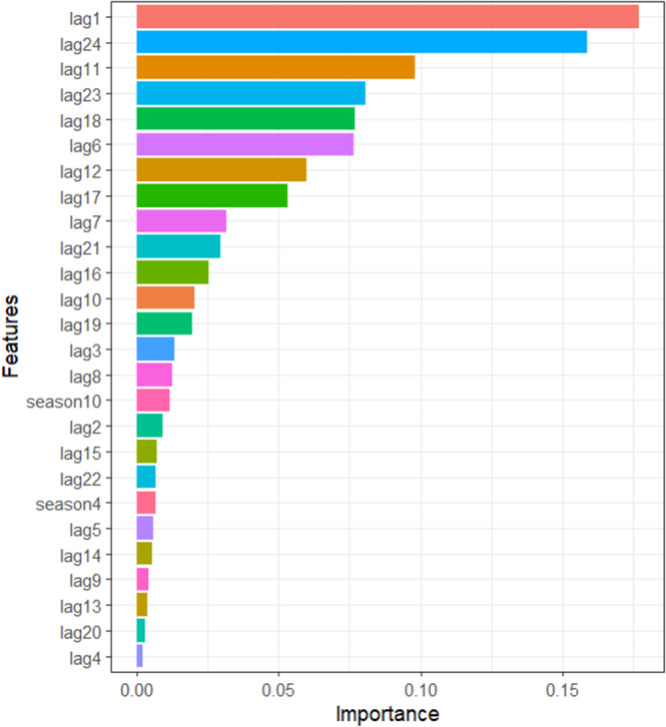
Key influential features of the XGBoost model.

According to the XGBoost model's feature significance analysis (Figure 7), recent dengue cases—especially those at lag 1—had the most impact on current incidence. Significant contributions were also made by longer‐term lags, including lags 11, 12, and 24, which showed patterns of recurrence over many years and annually. Because dengue transmission in Bangladesh is seasonal, seasonal factors had lower but consistent impacts.

**Figure 8:**
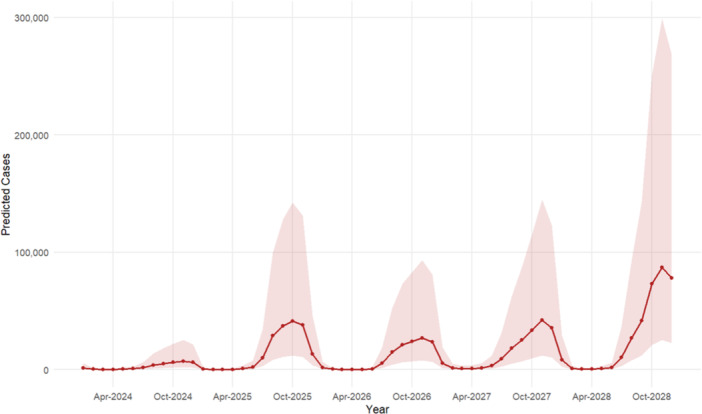
Five‐year‐ahead projection of monthly dengue cases with 95% prediction interval in Bangladesh using the XGBoost model.

Therefore, based on the superiority of the XGBoost model, the 5‐year‐ahead dengue cases were projected. The projections from the XGBoost model suggest that the number of dengue cases from 2024 to 2028 may range between 35,297 and 330,242 (Table S5 and Figure S4), under the assumption that historical transmission patterns persist. These projections indicate a significant upward trend in future dengue incidence (Figure 8). The forecasting plot shows pronounced seasonal peaks accompanied by widening 95% confidence intervals, reflecting greater uncertainty during high‐transmission months and over longer forecast horizons. These long‐term projections are conditional estimates that could be affected by future shifts in disease surveillance, intervention measures, or climate conditions.

## 4 Discussion

According to this study, although the monthly dengue incidence from 2000 to 2016 seems to remain relatively low, with several years reporting zero cases, a sharp increase was observed in 2017, followed by two major outbreaks in 2019 and 2023. Several factors could explain these peaks. Apart from favorable climatic conditions during the monsoon season—characterized by heavy rainfall and increased temperatures [31, 32] that promote mosquito breeding [33]—non‐climatic factors may also have contributed. These include improved surveillance systems, expanded laboratory testing capacity, and inadequate public awareness [34], leading to higher case reporting. Additionally, the possible introduction of new dengue virus serotypes or serotype shifts could have triggered larger outbreaks, as observed in previous years [8]. Rapid unplanned urbanization [34, 35], population growth, insufficient waste management, and poor drainage systems in densely populated cities like Dhaka [36, 37] further exacerbate the problem by providing additional breeding sites. Moreover, the incidence rates varied notably, remaining low from 2000 to 2016 and rising sharply after 2017, with peaks in 2019 and 2023. The lower rates may be due to underreporting or limited surveillance, while the higher rates likely reflect improved reporting and rapid urbanization. The seasonal trend of dengue cases exhibits a distinct pattern, with cases rising significantly beginning in June, peaking in August, and tapering off by October. This seasonal trend indicates that the middle of the year is particularly susceptible to outbreaks, likely due to favorable climatic conditions during the monsoon season. These insights emphasize the urgent necessity for public health authorities to anticipate surges in outbreaks and implement targeted control measures. Utilizing predictive modeling will be crucial to reducing future dengue risks in Bangladesh's rapidly evolving environment.

Unlike Bangladesh, Brazil shares similar climatic and urban challenges with Bangladesh, and has been at the forefront of applying machine learning (ML) techniques to dengue forecasting. For example, an ensemble ML technique [38] was used to estimate the dengue incidence rate in Brazil, taking into account climatic factors and focusing on the population under 19 years of age. Another study applied a Long Short‐Term Memory (LSTM) model [39] to predict weekly dengue incidence and outbreaks. These developments in Brazil highlight the potential of ML models to improve early warning systems and guide public health efforts, providing valuable lessons for Bangladesh.

In this context, the role of digital health tools and ML models becomes increasingly important. Digital health systems, such as real‐time surveillance platforms and mobile health (mHealth) applications [40], can significantly enhance predictive capabilities. Integrating ML models, particularly XGBoost, into these digital health tools can provide real‐time monitoring and decision support during outbreaks. For example, forecasting dengue incidences using XGBoost can be incorporated into mHealth platforms to provide public health authorities and vulnerable populations with timely information, potentially reducing the number of cases through early interventions. These tools could provide personalized health advice to at‐risk groups and inform public health campaigns. Although this research only examines past dengue case data, it establishes a foundation for future integration into early warning and digital health surveillance systems. When new data becomes available, the forecasting framework could be linked to current health information platforms to automatically generate warnings and updates. Such integration would enable dynamic monitoring, visualization of potential outbreak trajectories, and quick communication with communities and public health officials. Therefore, even though this study does not implement a digital or multi‐source EWS, its forecasts provide a valuable component that could be incorporated into future digital health systems to enhance Bangladesh's preparedness for dengue and for other low‐and middle‐income countries as well.

The ARIMA model incorporates trend components, seasonal information, cyclical factors, and random errors from the time series data. This model combines the advantages of autoregressive and moving average models, is not constrained by data sources, and is highly adaptable with strong short‐term predictive capabilities [30]. Rather than relying on specific influencing factors, the ARIMA model uses only historical data to understand disease patterns and generate more accurate forecasts. Consequently, the ARIMA approach is straightforward to learn and commonly employed. However, a drawback of the ARIMA model is that it cannot effectively capture nonlinear patterns. To address this limitation, ML techniques, such as eXtreme Gradient Boosting (XGBoost), provide an effective alternative. XGBoost is a powerful ensemble model built on decision tree techniques that maximizes computational efficiency and predictive performance [41, 42]. It is particularly useful for analyzing disease incidence data with complex patterns due to its exceptional ability to handle nonlinear relationships, manage large datasets, and accommodate missing values [24]. Moreover, XGBoost employs regularization strategies to mitigate overfitting, ensuring reliable and accurate predictions even in the presence of noise or high‐dimensional data [43]. One of the key advantages of XGBoost is its flexibility in hyperparameter tuning, as it allows for the regulation of more parameters compared to many alternative models [44]. By amalgamating many CART (Classification and Regression Trees) models, the XGBoost model can attain more generalizability compared to an individual model, indicating that XGBoost incurs a greater post‐pruning penalty than a GBDT (Gradient Boosted Decision Trees) model, hence rendering the trained model less susceptible to overfitting. In contrast to the intricate requirements of the ARIMA model, the modeling method of XGBoost is very straightforward. In this study, the ARIMA and XGBoost models were built using historical data from 2000 to 2023, after addressing seasonal adjustments, several orders, and transformations. The “auto.arima” function from the forecast package in R was used to generate a range of ARIMA models. The best ARIMA model was selected based on the Corrected Akaike's Information Criterion (AICc) values. The ARIMA(1,0,0) × (0,1,2)_12_ model was deemed significant. On the other hand, XGBoost was fitted by tuning several of its parameters, and the best model was selected based on exhibiting the lowest error. Finally, the two models were evaluated using the RMSE, MAE, and MASE metrics. The evaluation showed that XGBoost outperformed ARIMA, indicating it as the superior model. Therefore, using the XGBoost model, a projection of dengue incidence over the next 5 years (2024–2028) was made. The forecast suggests that the number of cases may range between 35,297 and 330,242, indicating a significant upward trend in future dengue incidences. It is crucial to recognize that due to modifications in case definitions, diagnostic procedures, or reporting capabilities over the study period, the dengue surveillance data utilized in this investigation may have structural breakdowns. Additionally, the COVID‐19 pandemic likely caused changes in healthcare‐seeking behavior and disruptions in reporting, especially between 2020 and 2021, which would have temporarily affected the overall number of reported cases. The dataset's temporal continuity and, in turn, the accuracy of long‐term projections may be impacted by several variables. The modeling methodology offers useful insights into general dengue patterns and their possible application in bolstering future surveillance and forecasting systems, notwithstanding these drawbacks. The prediction underscores the importance of strengthening early warning systems, integrating ML models into digital health platforms, and implementing more effective intervention measures to manage the anticipated increase in cases.

## 5 Strengths and Limitations

5.1

This study's primary strength lies in its use of XGBoost, a robust ML model adept at uncovering intricate, nonlinear patterns within dengue data. It surpassed ARIMA's performance, facilitating a 5‐year forecasting capability that aids in early warning systems, public health strategy, and digital health monitoring. Nevertheless, the model's dependence solely on historical case data means it overlooks socioeconomic and geographical factors that impact dengue transmission. Moreover, XGBoost faces limitations, such as its lower interpretability, the necessity for meticulous tuning, and reduced effectiveness in small‐sample situations. Future research should incorporate real‐time digital health information and explainable AI to boost the accuracy and relevance of forecasting.

## 6 Recommendation

Future studies should integrate real‐time digital health data with XGBoost forecasting, alongside explainable AI and prediction interval methods, to improve accuracy, interpretability, and the communication of forecast uncertainty for public health decision‐making.

## 7 Conclusion

This study meticulously analyzed historical dengue data and compared two forecasting models, ARIMA and XGBoost, to project incidence rates for the next 5 years. XGBoost emerged as the superior model due to its ability to capture intricate, nonlinear patterns. The analysis revealed a distinct seasonal pattern, with 2023 marking the highest recorded incidence. Looking ahead, projections indicate a significant and continued rise in dengue cases, which underscores the critical need for enhanced forecasting systems, especially those integrating digital health tools. Strengthening early warning systems, employing ML techniques, and implementing targeted interventions will be essential in managing the growing threat of dengue epidemics. These findings provide valuable insights for public health authorities and decision‐makers, urging proactive measures to mitigate the impact of future outbreaks and ensure more effective disease management strategies.

## Author Contributions

**Arman Hossain Chowdhury:** conceptualization, data curation, methodology, software, formal analysis, investigation, supervision, validation, visualization, project administration, resources, funding acquisition, writing – original draft, writing – review and editing.

## Funding

The author received no specific funding for this work.

## Ethics Statement

This study utilized publicly accessible, aggregated dengue case data from the Directorate General of Health Services (DGHS), Bangladesh. Since the dataset included no identifiable individual information, ethical approval and informed consent were not necessary.

## Conflicts of Interest

The author declares no conflicts of interest.

## Patient and Public Involvement

Patients and/or the public did not participate in the design, conduct, reporting, or dissemination of this research.

## Transparency Statement

The lead author Arman Hossain Chowdhury affirms that this manuscript is an honest, accurate, and transparent account of the study being reported; that no important aspects of the study have been omitted; and that any discrepancies from the study as planned (and, if relevant, registered) have been explained.

## Supporting information


**Figure S1:** The time series plot of the log‐transformed dengue data shows stationarity. **Figure S2:** Autocorrelation and partial autocorrelation plot. **Figure S3:** Actual, fitted, and forecasted dengue cases in Bangladesh as modeled by ARIMA and XGBoost. **Figure S4:** Actual vs. predicted (2024–2028) plot of dengue cases using xgboost model. **Table S1:** District‐wise incidence rates of dengue in Bangladesh in 2023. **Table S2:** Fitted ARIMA models and their Corrected Akaikes Information Criterion values. **Table S3:** Parameter tuning of the XGBoost model. **Table S4:** Performance measures in original scales for the test set. **Table S5:** Forecast of dengue cases in Bangladesh for the next 5 years (2024‐2028) with 95% prediction interval using XGBoost model.

## Data Availability

Detailed data and source codes are available at https://github.com/arman2018/bd-dengue-projection.
