# Supplementary material for: Data‐Driven Machine Learning–Based Forecasting of Dengue in Bangladesh: Supporting Digital Health Approaches for Early Warning
Source: Health Sci Rep. 2026 Mar 19;9(3):e72147. doi: 10.1002/hsr2.72147 (PMC13097490; doi:10.1002/hsr2.72147)
Supplement: Supplementary file 1 — Figure S1: The time series plot of the log‐transformed dengue data shows stationarity. Figure S2: Autocorrelation and partial autocorrelation plot. Figure S3: Actual, fitted, and forecasted dengue cases in Bangladesh as modeled by ARIMA and XGBoost. Figure S4: Actual vs. predicted (2024–2028) plot of dengue cases using xgboost model. Table S1: District‐wise incidence rates of dengue in Bangladesh in 2023. Table S2: Fitted ARIMA models and their Corrected Akaikes Information Criterion values. Table S3: Parameter tuning of the XGBoost model. Table S4: Performance measures in original scales for the test set. Table S5: Forecast of dengue cases in Bangladesh for the next 5 years (2024‐2028) with 95% prediction interval using XGBoost model. [file HSR2-9-e72147-s001.docx]

***Supplement***

**Data-Driven Machine Learning–Based Forecasting of Dengue in Bangladesh: Supporting Digital Health Approaches for Early Warning**

Arman Hossain Chowdhury

Department of Statistics, Begum Rokeya University, Rangpur, Bangladesh.

The study employed ARIMA and XGBoost to model the overall trend and compared the models on the basis of three prominent evaluation metrics, such as RMSE, MAE, and MASE, to identify the best model that can project the future dengue incidences in Bangladesh.

*ARIMA model*

ARIMA components are categorized into three parts: AR (autoregressive), I (integrated), and MA (moving average). The autoregressive order AR(p) represents a linear combination of observations from p previous time points, along with a random shock term. Mathematically, it can be expressed as:

$Y_{t}= C+\emptyset_{1}Y_{t-1}+\emptyset_{2} Y_{t-2}+\emptyset_{3} Y_{t -3}+\emptyset_{4} Y_{t-4} \ldots. .\emptyset_{p}Y_{t-p}+\varepsilon_{t}$ (1)

In this context, $Y_{t}$ and $\varepsilon_{t}$ represents the observed value and the random shock terms at time t. The parameter $\emptyset_{i}$ (where, i = 1,2,3,4....) signify the model parameters, and c is the constant term. Conversely, the moving average order MA(q) describes the dependence of the variable on previous random shock terms, which can be defined as:

$Y_{t}=\mu+\theta_{1}\varepsilon_{t-1}+\theta_{2}\varepsilon_{t-2}+\theta_{3}\varepsilon_{t-3}+\theta_{4}\varepsilon_{t-4}+\ldots+\theta_{q}\varepsilon_{t-q}+\varepsilon_{t}$ (2)

where, $\mu$ indicates the mean of the series, $\theta_{j}$ (j = 1, 2, 3... q) indicates the model parameters, and q denotes the model's order.

On the other hand, a seasonal ARIMA model captures information from seasonal components that a standard ARIMA model may not address. Seasonal models are categorized into two types based on their complexity: an additive model (simple seasonal model) and a multiplicative model. The mathematical expression for the simple seasonal model is:

$X_{t}=S_{t}+T_{t}+I_{t}$ (3)

where$S_{t}, T_{t}$ and $I_{t}$ represent seasonal information, trend information, and random fluctuations in the data, respectively.

*XGBoost model*

The objective function is

$Obj^{(t)}=\sum_{i=1}^{n} l\left( y_{i},\hat{y}_{i}^{\left( t-1 \right)}+f_{t}(x_{i}) \right)+\Omega\left( f_{t} \right)+constant$ (4)

Where $y_{i}$ is the observed count of dengue cases, $\hat{y}_{i}^{\left( t-1 \right)}$ is the estimated value from the prior iteration, $x_{i}$ is the input vector consists solely of lagged values from the dengue time series, n denotes the number of observations, $f_{t}$ denotes a distinct function that which algorithm trains, $\Omega\left( f_{t} \right)$ denotes the regularization term, which restrains models from overfitting. $l$ denotes the loss function, which computes the deviance between the label and the estimate in the earlier stage, the new tree's output.

To model the data, it was first observed that the data were not stable. So, a log transformation was applied to make the data stable and presented it in the following figure.

**
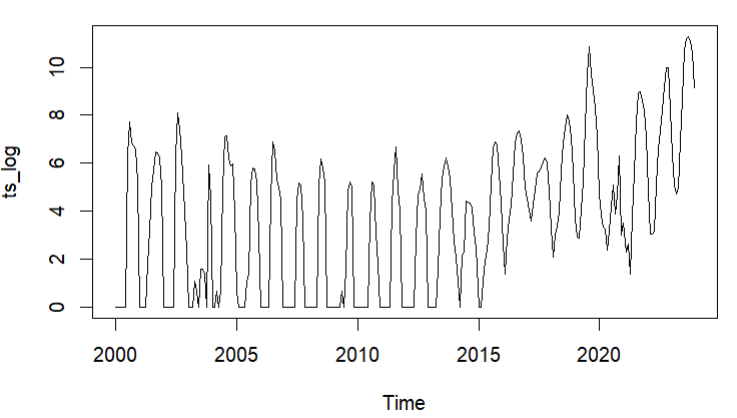
**

**Figure S1.** The time series plot of the log-transformed dengue data shows stationarity.


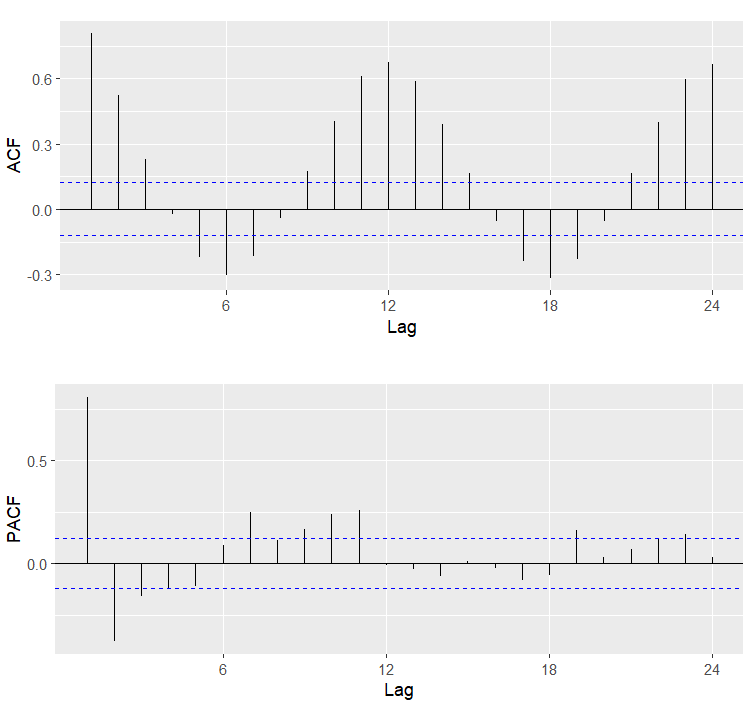


**Figure S2.** Autocorrelation and partial autocorrelation plot.


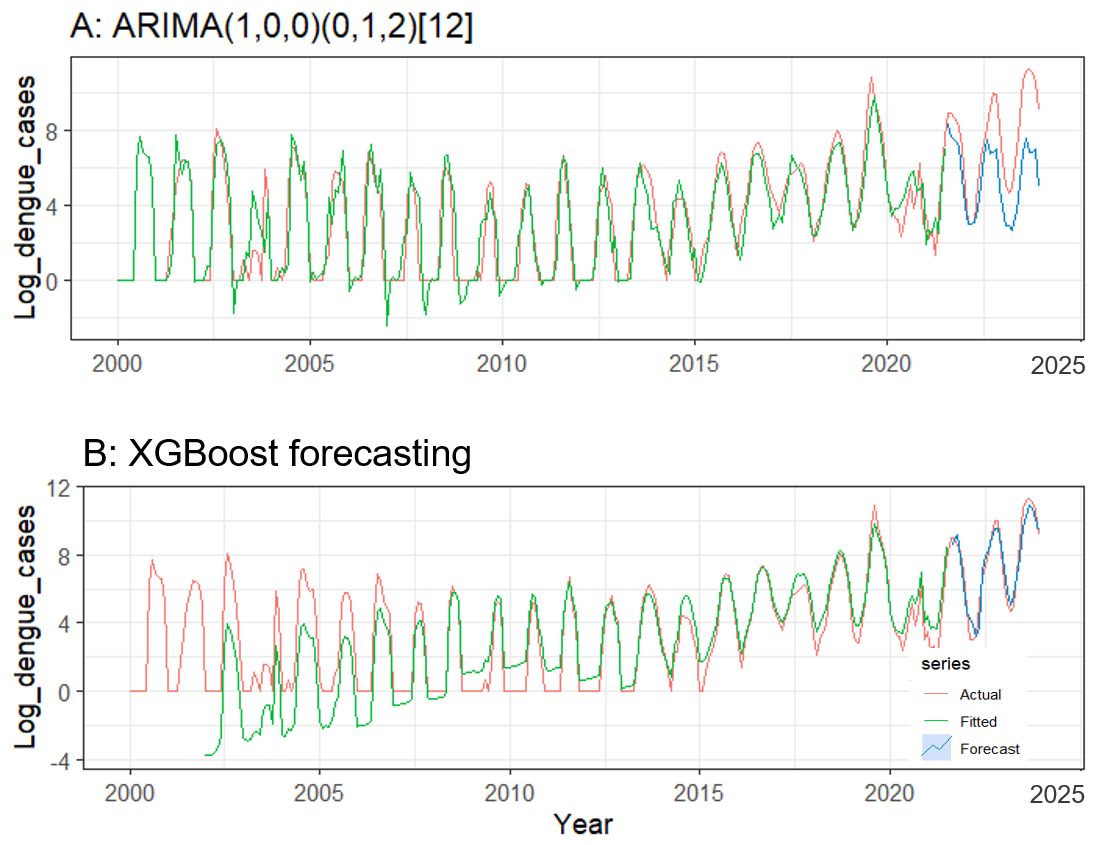


**Figure S3.** Actual, fitted, and forecasted dengue cases in Bangladesh as modeled by ARIMA and XGBoost.

| **Ljung-Box test** |
| --- |
|  |
| Data: Residuals from ARIMA(1,0,1) with non-zero mean |
| Q* = 1.5198, df = 22, p-value = 1 |
|  |
| Model df: 2. Total lags used: 24 |

**Table S1.** District-wise incidence rates of dengue in Bangladesh in 2023.

| Year | Divisions | Districts | Cases | Population | IR |
| --- | --- | --- | --- | --- | --- |
| 2023 | Dhaka | Dhaka | 113233 | 14734025 | 768.51 |
| 2023 | Dhaka | Faridpur | 7502 | 2162876 | 346.85 |
| 2023 | Dhaka | Gazipur | 7252 | 5263474 | 137.78 |
| 2023 | Dhaka | Gopalganj | 3156 | 1295053 | 243.70 |
| 2023 | Dhaka | Kishoregonj | 1549 | 3267630 | 47.40 |
| 2023 | Dhaka | Madaripur | 5386 | 1293027 | 416.54 |
| 2023 | Dhaka | Manikganj | 12952 | 1558024 | 831.31 |
| 2023 | Dhaka | Munshiganj | 2167 | 1625418 | 133.32 |
| 2023 | Dhaka | Narayanganj | 2205 | 3909138 | 56.41 |
| 2023 | Dhaka | Narsingdi | 4523 | 2584452 | 175.01 |
| 2023 | Dhaka | Rajbari | 4176 | 1189821 | 350.98 |
| 2023 | Dhaka | Shariatpur | 2901 | 1294561 | 224.09 |
| 2023 | Dhaka | Tangail | 2319 | 4037608 | 57.43 |
| 2023 | Mymensingh | Mymensingh | 4533 | 5899052 | 76.84 |
| 2023 | Mymensingh | Jamalpur | 1692 | 2499737 | 67.69 |
| 2023 | Mymensingh | Sherpur | 1281 | 1501853 | 85.29 |
| 2023 | Mymensingh | Netrokona | 762 | 2324856 | 32.78 |
| 2023 | Chittagong | Chittagong | 14200 | 9169464 | 154.86 |
| 2023 | Chittagong | Cox's Bazar | 4659 | 2823265 | 165.02 |
| 2023 | Chittagong | Bandarban | 739 | 481109 | 153.60 |
| 2023 | Chittagong | Rangamati | 491 | 647587 | 75.82 |
| 2023 | Chittagong | Khagrachari | 827 | 714119 | 115.81 |
| 2023 | Chittagong | Feni | 2228 | 1648896 | 135.12 |
| 2023 | Chittagong | Noakhali | 1985 | 3625252 | 54.75 |
| 2023 | Chittagong | Cumilla | 5387 | 6212216 | 86.72 |
| 2023 | Chittagong | Chandpur | 5536 | 2635748 | 210.04 |
| 2023 | Chittagong | Lakshmipur | 6950 | 1938111 | 358.60 |
| 2023 | Chittagong | Brahmanbariah | 1433 | 3306559 | 43.34 |
| 2023 | Khulna | Khulna | 6853 | 2613385 | 262.23 |
| 2023 | Khulna | Bagerhat | 1720 | 1613079 | 106.63 |
| 2023 | Khulna | Satkhira | 1500 | 2196581 | 68.29 |
| 2023 | Khulna | Jessore | 4974 | 3076849 | 161.66 |
| 2023 | Khulna | Jhenaidah | 4194 | 2005849 | 209.09 |
| 2023 | Khulna | Magura | 5031 | 1033115 | 486.97 |
| 2023 | Khulna | Narail | 2841 | 788673 | 360.23 |
| 2023 | Khulna | Kushtia | 4556 | 2149692 | 211.94 |
| 2023 | Khulna | Chuadanga | 877 | 1234066 | 71.07 |
| 2023 | Khulna | Meherpur | 2176 | 705356 | 308.50 |
| 2023 | Rajshahi | Rajshahi | 6078 | 2915013 | 208.51 |
| 2023 | Rajshahi | Chapai-nawabganj | 1448 | 1835527 | 78.89 |
| 2023 | Rajshahi | Naogaon | 740 | 2784598 | 26.57 |
| 2023 | Rajshahi | Natore | 1063 | 1859921 | 57.15 |
| 2023 | Rajshahi | Joypurhat | 264 | 956430 | 27.60 |
| 2023 | Rajshahi | Bagura | 2057 | 3734300 | 55.08 |
| 2023 | Rajshahi | Sirajganj | 4371 | 3357708 | 130.18 |
| 2023 | Rajshahi | Pabna | 3388 | 2909622 | 116.44 |
| 2023 | Rangpur | Rangpur | 1299 | 3169615 | 40.98 |
| 2023 | Rangpur | Lalmonirhat | 305 | 1428406 | 21.35 |
| 2023 | Rangpur | Kurigram | 708 | 2329161 | 30.40 |
| 2023 | Rangpur | Nilphamari | 770 | 2092567 | 36.80 |
| 2023 | Rangpur | Dinajpur | 910 | 3315238 | 27.45 |
| 2023 | Rangpur | Gaibandha | 1052 | 2562232 | 41.06 |
| 2023 | Rangpur | Thakurgaon | 309 | 1533894 | 20.14 |
| 2023 | Rangpur | Panchagarh | 187 | 1179843 | 15.85 |
| 2023 | Barisal | Barisal | 13603 | 2570450 | 529.21 |
| 2023 | Barisal | Patuakhali | 7579 | 1727254 | 438.79 |
| 2023 | Barisal | Bhola | 3861 | 1932514 | 199.79 |
| 2023 | Barisal | Pirojpur | 7361 | 1198193 | 614.34 |
| 2023 | Barisal | Barguna | 4592 | 1010530 | 454.42 |
| 2023 | Barisal | Jhalokathi | 1053 | 661161 | 159.27 |
| 2023 | Sylhet | Sylhet | 711 | 3857037 | 18.43 |
| 2023 | Sylhet | Sunamganj | 102 | 2695495 | 3.78 |
| 2023 | Sylhet | Habiganj | 493 | 2358886 | 20.90 |
| 2023 | Sylhet | Maulvibazar | 129 | 2123445 | 6.08 |
| 2023 | National | | 321179 | 165158616 | 194.47 |

**Table S2.** Fitted ARIMA models and their Corrected Akaikes Information Criterion values

| **Fitted Models** | **AICc values** |
| --- | --- |
| ARIMA(2,0,2)(0,1,1)[12] with drift | 822.258 |
| ARIMA(0,0,0)(0,1,0)[12] with drift | 1070.409 |
| ARIMA(1,0,0)(0,1,0)[12] with drift | 936.775 |
| ARIMA(0,0,1)(0,1,1)[12] with drift | 870.438 |
| ARIMA(0,0,0)(0,1,0)[12] | 1068.477 |
| ARIMA(2,0,2)(0,1,0)[12] with drift | 941.983 |
| ARIMA(2,0,2)(0,1,2)[12] with drift | 819.443 |
| ARIMA(1,0,2)(0,1,2)[12] with drift | 820.657 |
| ARIMA(2,0,1)(0,1,2)[12] with drift | 820.773 |
| ARIMA(3,0,2)(0,1,2)[12] with drift | Inf |
| ARIMA(2,0,3)(0,1,2)[12] with drift | 821.319 |
| ARIMA(1,0,1)(0,1,2)[12] with drift | 818.680 |
| ARIMA(1,0,1)(0,1,1)[12] with drift | 821.934 |
| ARIMA(0,0,1)(0,1,2)[12] with drift | 863.130 |
| ARIMA(1,0,0)(0,1,2)[12] with drift | 816.929 |
| ARIMA(1,0,0)(0,1,1)[12] with drift | 820.198 |
| ARIMA(0,0,0)(0,1,2)[12] with drift | 967.559 |
| ARIMA(2,0,0)(0,1,2)[12] with drift | 818.703 |
| ARIMA(1,0,0)(0,1,2)[12] | 816.076 |
| ARIMA(1,0,0)(0,1,1)[12] | 820.400 |
| ARIMA(0,0,0)(0,1,2)[12] | 967.423 |
| ARIMA(2,0,0)(0,1,2)[12] | 817.918 |
| ARIMA(1,0,1)(0,1,2)[12] | 817.898 |
| ARIMA(0,0,1)(0,1,2)[12] | 863.298 |
| ARIMA(2,0,1)(0,1,2)[12] | 819.967 |

**Table S3.** Parameter tuning of the XGBoost model

| **nrounds** | **Lambda** | **nfold** | **Trend** | **RMSE** | |
| --- | --- | --- | --- | --- | --- |
|  |  |  |  | **Train** | **Test** |
| 23 | 1 | 10 | *differencing* | 1.53 | 0.63 |
| 24 | 1 | 10 | *differencing* | 2.21 | 0.75 |
| 25 | 1 | 10 | *differencing* | 4.45 | 1.94 |
| 26 | 1 | 10 | *differencing* | 5.34 | 1.16 |
| 27 | 1 | 10 | *differencing* | 2.21 | 0.75 |

**Table S4.** Performance measures in original scales for test set

| Error metrics | ARIMA | XGBoost |
| --- | --- | --- |
| RMSE | 26315.35 | 11770.43 |
| MAE | 13367.51 | 5866.35 |
| MASE | 0.51 | 0.50 |

**Table S5.** Forecast of dengue cases in Bangladesh for the next 5 years (2024-2028) with 95% prediction interval using XGBoost model.

| Year | Months | Prediction | Lower 95% | Upper 95% | Yearly total |
| --- | --- | --- | --- | --- | --- |
| 2024 | Jan | 1612.16 | 469.13 | 5554.86 | 35297.88 |
| 2024 | Feb | 451.73 | 131.57 | 1565.65 |  |
| 2024 | Mar | 308.73 | 89.97 | 1074.06 |  |
| 2024 | Apr | 296.57 | 86.43 | 1032.27 |  |
| 2024 | May | 596.28 | 173.62 | 2062.59 |  |
| 2024 | Jun | 820.15 | 238.74 | 2832.18 |  |
| 2024 | Jul | 1856.16 | 540.11 | 6393.64 |  |
| 2024 | Aug | 4054.12 | 1179.48 | 13949.57 |  |
| 2024 | Sep | 5275.16 | 1534.67 | 18147.13 |  |
| 2024 | Oct | 6372.30 | 1853.82 | 21918.74 |  |
| 2024 | Nov | 7379.94 | 2146.94 | 25382.70 |  |
| 2024 | Dec | 6274.57 | 1825.39 | 21582.76 |  |
| 2025 | Jan | 640.20 | 186.39 | 2213.57 | 173731.03 |
| 2025 | Feb | 172.01 | 50.20 | 604.06 |  |
| 2025 | Mar | 182.34 | 53.20 | 639.57 |  |
| 2025 | Apr | 361.06 | 105.19 | 1253.97 |  |
| 2025 | May | 972.40 | 283.03 | 3355.57 |  |
| 2025 | Jun | 2272.36 | 661.18 | 7824.40 |  |
| 2025 | Jul | 9957.57 | 2896.75 | 34243.79 |  |
| 2025 | Aug | 28929.64 | 8415.59 | 99463.91 |  |
| 2025 | Sep | 37219.39 | 10827.02 | 127961.51 |  |
| 2025 | Oct | 41370.83 | 12034.64 | 142232.86 |  |
| 2025 | Nov | 38139.48 | 11094.67 | 131124.50 |  |
| 2025 | Dec | 13513.75 | 3931.22 | 46468.83 |  |
| 2026 | Jan | 2027.17 | 589.85 | 6981.53 | 120773.21 |
| 2026 | Feb | 410.79 | 119.66 | 1424.91 |  |
| 2026 | Mar | 273.22 | 79.64 | 952.00 |  |
| 2026 | Apr | 239.53 | 69.84 | 836.19 |  |
| 2026 | May | 297.86 | 86.81 | 1036.70 |  |
| 2026 | Jun | 624.63 | 181.86 | 2160.04 |  |
| 2026 | Jul | 5570.60 | 1620.61 | 19162.76 |  |
| 2026 | Aug | 15233.31 | 4431.43 | 52380.16 |  |
| 2026 | Sep | 21296.25 | 6195.09 | 73222.67 |  |
| 2026 | Oct | 24125.60 | 7018.13 | 82949.10 |  |
| 2026 | Nov | 27147.86 | 7897.28 | 93338.68 |  |
| 2026 | Dec | 23526.38 | 6843.82 | 80889.17 |  |
| 2027 | Jan | 5562.26 | 1618.19 | 19134.08 | 178572.76 |
| 2027 | Feb | 1546.73 | 450.09 | 5329.91 |  |
| 2027 | Mar | 1065.95 | 310.24 | 3677.14 |  |
| 2027 | Apr | 1073.04 | 312.30 | 3701.52 |  |
| 2027 | May | 1601.47 | 466.02 | 5518.12 |  |
| 2027 | Jun | 3539.59 | 1029.81 | 12180.77 |  |
| 2027 | Jul | 9110.00 | 2650.20 | 31330.10 |  |
| 2027 | Aug | 18194.90 | 5292.93 | 62561.17 |  |
| 2027 | Sep | 25407.11 | 7390.91 | 87354.55 |  |
| 2027 | Oct | 33543.80 | 9757.82 | 115325.96 |  |
| 2027 | Nov | 42116.69 | 12251.61 | 144796.90 |  |
| 2027 | Dec | 35811.23 | 10417.40 | 123120.68 |  |
| 2028 | Jan | 8468.32 | 2463.54 | 29124.21 | 330242.92 |
| 2028 | Feb | 1044.48 | 304.00 | 3603.35 |  |
| 2028 | Mar | 438.70 | 127.78 | 1520.85 |  |
| 2028 | Apr | 455.19 | 132.57 | 1577.55 |  |
| 2028 | May | 867.21 | 252.43 | 2993.94 |  |
| 2028 | Jun | 1699.76 | 494.61 | 5855.98 |  |
| 2028 | Jul | 10615.58 | 3088.16 | 36505.83 |  |
| 2028 | Aug | 27017.82 | 7859.46 | 92891.66 |  |
| 2028 | Sep | 41684.59 | 12125.92 | 143311.49 |  |
| 2028 | Oct | 72995.06 | 21233.91 | 250947.22 |  |
| 2028 | Nov | 87002.99 | 25308.72 | 299102.15 |  |
| 2028 | Dec | 77953.21 | 22676.20 | 267991.80 |  |


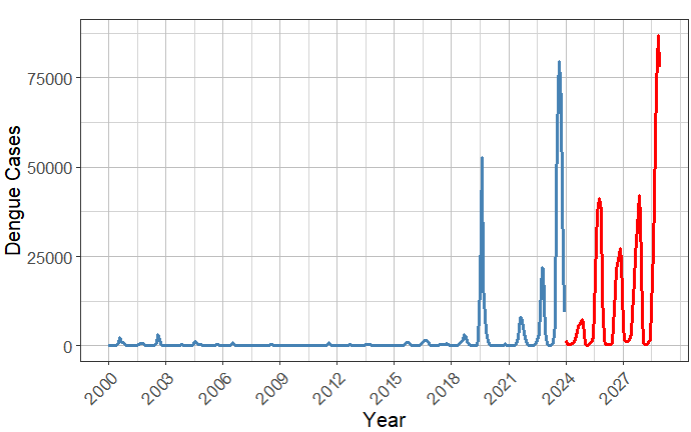


**Figure S4.** Actual vs. predicted (2024-2028) plot of dengue cases using xgboost model.
